# Characterization of serum metabolome and respiratory microbiota in children with influenza A virus infection

**DOI:** 10.3389/fcimb.2024.1478876

**Published:** 2025-01-30

**Authors:** Xinyi Shi, Shenghao Hua, Zeyuan Chen, Weiyi Cao, Mengqing Xiao, Wenlong Pei, Zhe Cao, Zhan Zhang, Haibing Yang, Xuejun Shao, Yu Xia

**Affiliations:** ^1^ Center for Global Health, School of Public Health, Nanjing Medical University, Nanjing, China; ^2^ Department of Clinical Laboratory, Children’s Hospital of Soochow University, Suzhou, China; ^3^ SCIex Analytical Instrument Trading Co., Ltd, Shanghai, China; ^4^ The First School of Clinical Medicine, Nanjing Medical University, Nanjing, China; ^5^ Suzhou Center for Disease Control and Prevention, Suzhou, China; ^6^ Suzhou College, Nanjing Medical University, Suzhou, China

**Keywords:** influenza A virus, children, respiratory tract, microbiota, metabolome

## Abstract

The risk of children being infected with Influenza A virus (IAV) is high, and if not treated promptly, it can lead to serious illness. Compared with control group, IAV infection decreased the contents of platelet, white blood cell, lymphocyte, eosinophil, basophil, CD3^+^ T cells, CD4^+^ T cells, CD8^+^ T cells, and B cells, while increasing the number of red blood cell. Additionally, IAV infection increased serum concentrations of total protein, albumin and lipase, while decreasing the contents of calcium, triglyceride, total bilirubin, direct bilirubin, indirect bilirubin and gamma-glutamyltransferase. However, the interactions between the respiratory microbiome and metabolites and their impact on IAV in children remains unclear. Ultra performance liquid chromatography quadrupole time of flight mass spectrometry (UPLC-QTOF/MS) and 16S rRNA gene sequencing were employed to analysis the respiratory microbiome and serum metabolic characteristics of 85 patients with IAV infection and age-matched 55 controls with respiratory disease who tested negative for 13 types of respiratory pathogens. The serum metabolic profile of IAV patients was significantly changed, and the purine metabolism was destroyed. Purine metabolism was also enriched in H3N2 patients compared to H1N1, with increased xanthine, deoxyguanosine, and inosine. The respiratory microbiome structure in children with IAV, including H1N1 and H3N2, was significantly different from that of the control, with significantly increased Chao index. The Mantel test revealed the correlation and consistency in the trends of *Haemophilus*, *Ureaplasma* and Inosine. This study revealed the characteristics of the respiratory microbiome and serum metabolites in pediatric patients with IAV, providing a new direction for exploring the pathogenesis of IAV in children.

## 1 Introduction

Influenza virus is an infectious respiratory pathogen that encompasses four types: influenza A virus (IAV), IBV, ICV and IDV (Uyeki, 2021; Wolf and Antoon, 2023). Among them, IAV is primarily responsible for clinical illness (Ciminski et al., 2021). Symptoms of influenza infection confined to the upper respiratory tract (URT) typically include fever, sore throat, runny nose, cough, nasal congestion, and throat discomfort. However, severe cases can lead to fatal pneumonia or secondary bacterial infections of the lower respiratory tract (LRT). In addition, influenza can result in respiratory complications, such as cardiac and central nervous system diseases (Sellers et al., 2017; Kwong et al., 2018). Influenza is characterized by annual seasonal epidemics, during which acquired immunity may wane over time following infection (Saad-Roy et al., 2019; Lam et al., 2020). However, when a novel strain emerges from animals and gains the ability to transmit efficiently among humans, a pandemic ensues (Hu et al., 2023). These pandemic strains are antigenically distinct from previous circulating strains, leading to more and the lack of immunity in humans leading to a more severe infection and increased mortality rate.

IAV can be categorized into various subtypes based on specific combinations of its surface molecules hemagglutinin (HA) and neuraminidase (NA). There are 16 HA isoforms (H1-H16) and 9 NA isoforms (N1-N9) (Dominoni et al., 2022; Murthy et al., 2023). Among these viruses, H1N1, H2N2 and H3N2 primarily infect humans, with H1N1 and H3N2 being the main subtypes causing seasonal influenza. Historically, six world pandemics have been predominantly caused by these two subtypes. Influenza infection is prevalent among children, particularly those under 5 years of age. The reported incidence of influenza in this age group is approximately 900,000 cases per year (Wang et al., 2021). Young children account for the majority of patients seeking influenza-related care, with a hospitalization rate of 1,000 per 100,000 people annually for children under 5 years old10. Globally, it is estimated that nearly 28,000 children under 18 years old die from influenza-related LRT infections each year (Antoon et al., 2024).

At present, there remains a significant gap in our understanding of how respiratory microbiota and serum metabolites influence inflammatory response and immune function in pediatric patients infected by influenza virus. The serum metabolome encompasses a plethora of biomarkers those are highly heritable (Collaborators G.B.D.I, 2019; Zeng et al., 2024) or influenced by the microbiome (Long et al., 2017). Therefore, integrated analysis of microorganisms and metabolites is of great value in exploring the potential etiology of diseases. Previous studies have indicated that infections can induce changes in relevant metabolites and alter microbial structure, potentially influencing disease progression and exacerbating respiratory disease (Li et al., 2024). Microbiota plays a crucial role in generating metabolites and participating in the physiological and pathological processes of the host (Mendez et al., 2019). In the context of IAV infection, the virus may disrupt cellular metabolic pathways by directly or indirectly stimulating the host immune system (Tian et al., 2023).

Current researches on influenza has primarily utilized cell culture or animal models, leaving the relevance of interactions between the respiratory microbiome and serum metabolites to susceptibility to IAV infection and disease severity in children poorly understood. Therefore, this study aimed to analyze the interaction of respiratory microbiome and serum metabolome in the etiology of IAV infection in children. This study will offer new insights into the etiology of IAV in children and provide valuable information for early risk prediction.

## 2 Materials and methods

### 2.1 Detection of influenza A virus

The sputum samples were used for pathogen testing by one-step RT-PCR using multiple respiratory pathogen detection kit (Health gene technologies, China) comprising 13 types of pathogens, including Influenza A virus (H7N9, H1N1, H3N2 and H5N2), influenza A virus H1N1 (2009), seasonal H3N2 virus, influenza B virus (Victoria strain and Yamagata strain), adenovirus (group B, group C and E), human bocavirus, rhinovirus, parainfluenza virus (type 1, 2, 3 and 4), coronavirus (type 229E, OC43, NL63 and HKU1), respiratory syncytial virus (Group A and B), metapneumovirus, Mycoplasma pneumoniae, and Chlamydia (Chlamydia trachomatis and Chlamydia pneumoniae).

### 2.2 Study participants

Pediatric patients admitted to Suzhou Children’s Hospital were enrolled and categorized into two groups: IAV group and control group. In this study, patients met the following criteria: 1) presence of relevant epidemiological history and clinical symptoms; 2) confirmation of IAV positivity using a combination of RT-PCR and capillary electrophoresis. The control group comprised pediatric patients who tested negative for 13 types of pathogens. Exclusion criteria encompassed: 1) Pre-existing diseases affecting the respiratory microbiota and metabolome, such as asthma and cystic fibrosis; 2) use of medications prior to enrollment known to impact the respiratory microbiome and metabolome, such as immunosuppressants, probiotics, and hormones; 3) pediatric patients who declined laboratory tests after admission. A total of 85 IAV cases and 55 control were enrolled, with an average age of about 4 years. There no significant differences in age, gender or related clinical diagnosis between the two groups (**Supplementary Table S1**). This study was approved by the Ethics Committee of Children’s Hospital of Soochow University (Approval No.2023 C143).

### 2.3 Respiratory samples collections

Respiratory specimens from the upper respiratory tract (URT) and the lower respiratory tract (LRT) were collected from IAV and control groups within 24 hours after admission. During the collection of specimens from URT, 5 mL normal saline was injected into one nostril with a pipette and the washings were collected in a dish or beaker. Due to the young age of children, it is difficult to obtain LRT specimens by alveolar lavage. The importance of oral cleanliness and deep cough were fully explained to the patients to avoid oropharyngeal bacterial contamination, and the patients were instructed how to correctly collect sputum specimens. The first sputum from pediatric patients after repeated gargle with water were collected covered to avoid the spread of microbiota. The collected specimens were immediately sent for IAV analysis or stored at -80°C for further microbiota analysis.

### 2.4 Blood routine test, blood biochemical test and flow cytometry

Fasting venous blood were collected on the morning after admission. Well-mixed anticoagulated whole blood was collected for blood routine tests using an Automatic Blood Cell analyzer BC-7500CRP. After erythrocytes were lysed with BD FACS hemolysin, the lymphocytes were stained with the lymphocyte subgroup detection Reagent (BD Multitest 6- Color TBNK Reagent), including CD3-FITC, CD4-PE-Cy7, CD8-APC-Cy7, CD56-PE, CD16-PE, CD19-APC, CD45-PerCP-Cy5.5, and analyzed by flow cytometry (BD FACSLyric). A Biochemical Analyzer ADVIA 2400 was employed to analyze the serum chemistry.

### 2.5 16S rRNA sequencing

Total DNA was extracted from the collected respiratory samples. The quality, concentration and purity of DNA were detected by 1% agarose gel electrophoresis and instruments. 27F (5’-AGAGTTTGATCCTGGCTCAG-3’) and 1492R (5’-GGTTACCTTGTTACGACTT-3’) were used to amplify the full-length 16S rRNA gene. PCR products were purified by DNA gel recovery and purification kit (Majorbio, China) and quantified by Synergy HTX (Majorbio, China). Amplicon libraries were confirmed using the NEXTFLEX Rapid DNA-Seq Kit (Bioo Scientific, USA) prior to sequencing eligible libraries. Sequencing was performed using Illumina’s MiSeq (PE300).

Quality control of the original sequencing data was conducted using fastp (https://github.com/OpenGene/fastp, version 0.20.0), while FLASH (http://www.cbcb.umd.edu/software/flash, version 1.2.11) was employed for sequence merging. Noise reduction of optimized sequences after quality control was performed using the divisive amplicon denoising algorithm 2 (DADA2) in the Qiime2 pipeline, resulting in amplicon sequence variants (ASVs). To minimize the impact of sequencing depth on subsequent analyses of alpha and beta diversity, all sample sequences were rarefied to 10,000. Taxonomic classification of ASVs was performed using the Naive Bayes classifier in Qiime2, referencing the Silva 16S rRNA gene database (v138). Subsequently, the data was analyzed on the online platform of Majorbio (www.majorbio.com). Phylogenetic Investigation of Communities by Reconstruction of Unobserved States 2 (PICRUSt2) was used to predict the functional information of microbial communities, and Kyoto Encyclopedia of Genes and Genomes (KEGG) combined with STAMP software was used to analyze the species and functional composition and differences.

### 2.6 Serum metabolomes and data processing

The SCIEX ZenoTOF 7600 system was employed to analyze serum metabolic profiling. The analytical column used was a Phenomenex F5 (2.1×100 mm, 1.8, Phenomenex, Castel Maggiore, Italy), housed in a compartment maintained at 45°C ± 1°C.

The autosampler temperature was maintained 10°C with an injection volume of 2 µL. Between each sample injection, the autosampler syringe was flushed for 5 s with a 2:1:1 (v/v) solution composed of water, methanol, and isopropyl alcohol. The gradient program was as follows: 0% B for 0–2 min, increased to 95% B from 2–14 min, held at 95% B from 14–16 min, decreased to 0% B from 16–16.1 min, and maintained at 0% B from 16.1–20 min to re-equilibrate the column. Electrospray ionization (ESI) was operated in both positive and negative ion modes with the following parameters: spray voltage +5 kV (positive) and -4.5 kV (negative), capillary temperature 550°C, gas1 50, gas2 50, and curtain gas 25. Data acquisition was performed in full scan mode (m/z 60–1200 Da) with an accumulation time of 0.2 s for MS1 and 15 ms for MS2 (m/z 3–1200 Da).

The stability of the instrument was checked by quality control samples (QC). Simca14.0 software was used for systematic analysis, such as orthogonal partial least squares discriminant analysis (OPLS-DA), to obtain variable weight importance ranking (VIP). The statistically significant differential metabolites were identified according to VIP> 1, P value< 0.05, and fold change> 1.2. The metabolic pathways were annotated using MetaboAnalyst 6.0 (www.metaboanalyst.ca) and KEGG database.

### 2.7 Statistical analysis

The data were analyzed by R software, and the comparison between the two groups was performed by unpaired two-tailed t test. Wilcox rank sum test was used to compare the difference of bacterium between the two groups. Correlations between metagenomics and metabolomics data were analyzed using the Mantel test and Spearman’s rank test. All data are presented as mean± SEM unless otherwise stated. P< 0.05 was considered statistically significant.

## 3 Result

### 3.1 Effects of IAV infection on blood cells

**Figure 1:**
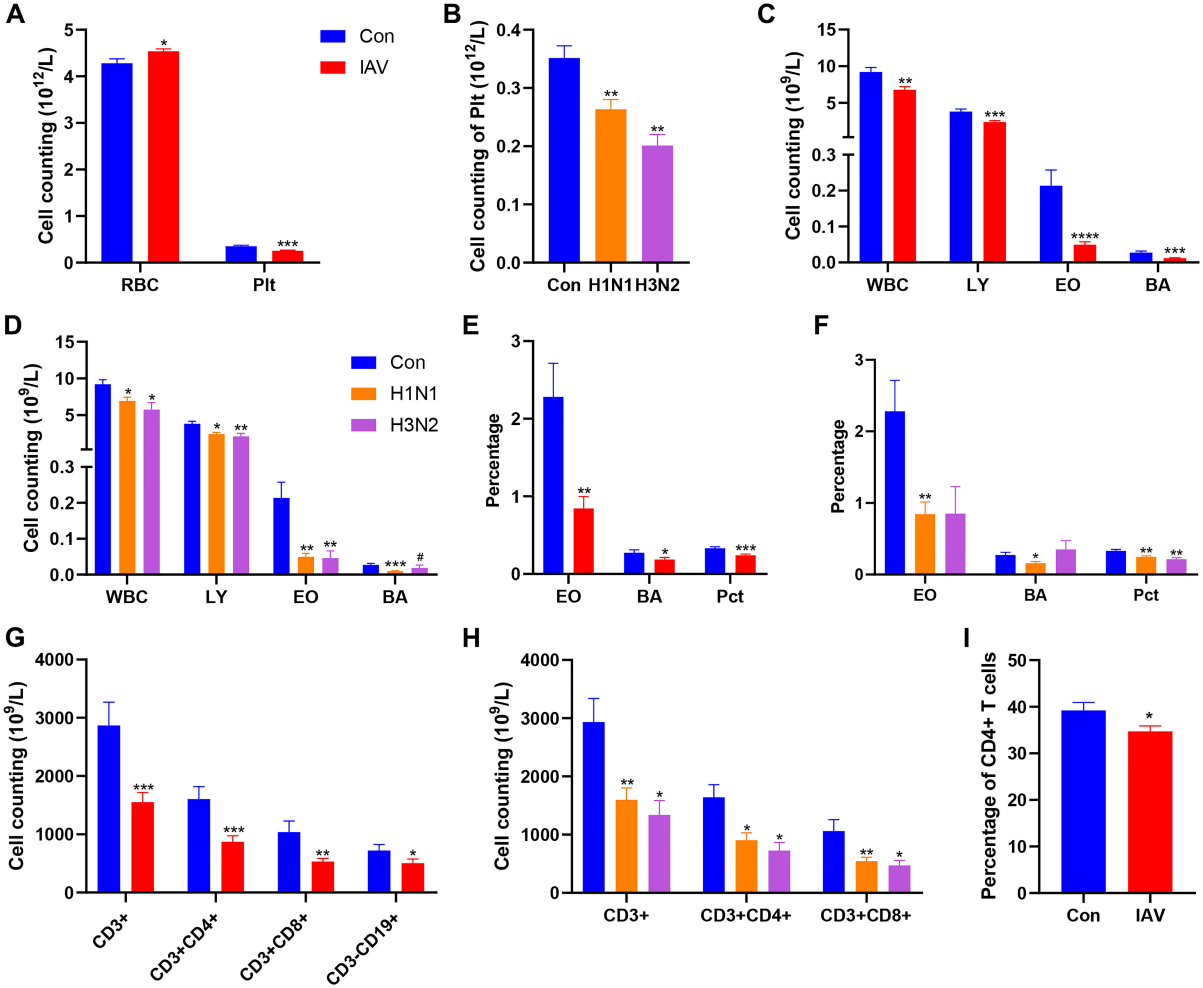
Effects IAV infection on blood cells. among Con, IAV, H1N1 and H3N2 groups. **(A)** The number of RBC and Plt between Con and IAV groups. **(B)** The cell counting of Plt among Con, H1N1 and H3N2 groups. **(C)** Cell counting of WBC, LY, EO and BA between Con and IAV groups. **(D)** Cell counting of WBC, LY, EO and BA among Con, H1N1 and H3N2 groups. **(E)** Percentages of EO, BA and Pct between Con and IAV groups. **(F)** Percentages of EO, BA and Pct among Con, H1N1 and H3N2 groups. **(G)** Cell counting of CD3+ T cells, CD3+CD4+ T cells, CD3+CD8+ T cells and CD3-CD19+ B cells between Con and IAV groups. **(H)** Cell counting of CD3+ T cells, CD3+CD4+ T cells and CD3+CD8+ T cells among Con, H1N1 and H3N2 groups. **(I)** The percentage of CD3+CD4+ T cells in the Con and IAV groups. Data was presented as the mean± SEM and analyzed by unpaired two-tailed t test. *P< 0.05, **P< 0.01, ***P< 0.001, ****P<0.0001, compared with Con group. #*P*< 0.05, compared with H1N1 group.

Compared with the control group, the children with IAV infection showed a significantly higher red blood cell (RBC) count and a markedly lower platelet (Plt) count. This reduction in Plt count was also observed within the H1N1 and H3N2 subtypes (**Figures 1A, B**). White blood cells (WBC), lymphocytes (LY), eosinophils (EO) and basophils (BA) counts were decreased after IAV infection, including H1N1 and H3N2 (**Figures 1C, D**). The percentage of plateletcrit (Pct) was decreased after both H1N1 and H3N2 infection, the proportion of EO and BA was decreased after IAV infection, especially H1N1 (**Figures 1E, F**). The number of B cells (CD3^-^CD19^+^) was decreased in IAV group T cells (CD3^+^), CD4^+^ T cells, CD8^+^ T cells were significantly decreased in both H1N1 and H3N2 subgroups (**Figures 1G, H**). FCM analysis also revealed the significant reduction of CD4^+^ T cells in IVA-infected patients (**Figure 1I**). After IAV infection, children with bronchopneumonia (BPI) have higher counts of BA, Plt and CD8^+^ T cell (**Supplementary Figure S1A**) and percentage of Pct, T cells and CD4^+^ T cells (**Supplementary Figures S1B-D**) than those with URT infections (UI). Compared with UI group, NK cells (CD3^-^CD(16 + 56)^+^) were increased in BPI group (**Supplementary Figure S1E**). Although, IAV infection failed to change the concentration of CRP, IAV-infected boys have lower CRP than girls (**Supplementary Figure S1F**). The concentrations of NE, MO, Hgb, Hct, MCV, MCH, MCHC, RDW, MPV, PDW, CD4^+^CD8^+^ and CD19^+^CD23^+^ in IAV patients were not statistically different from those of the control group (**Supplementary Tables S2-S5**).

### 3.2 Effects of IAV infection on serum chemistry

**Figure 2:**
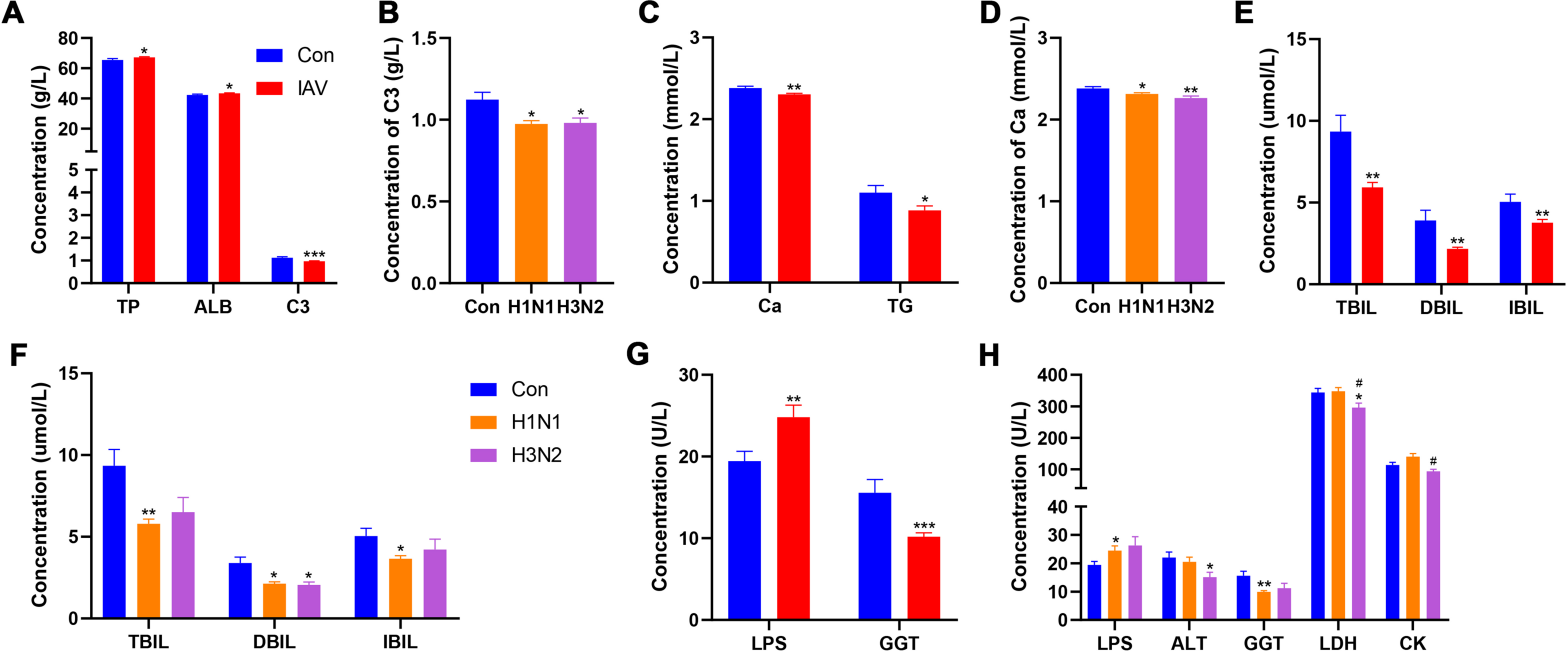
Effects IAV infection on serum chemistry. **(A)** Concentrations of TP, ALB and C3 between Con and IAV groups. **(B)** The concentration of C3 among Con, H1N1 and H3N2 groups. **(C)** Concentrations of Ca and TG between Con and IAV groups. **(D)** The concentration of Ca among Con, H1N1 and H3N2 groups. **(E)** Concentrations of TBIL, DBIL and IBIL between Con and IAV groups. **(F)** Concentrations of TBIL, DBIL and IBIL among Con, H1N1 and H3N2 groups. **(G)** Concentrations of LPS and GGT between Con and IAV groups. **(H)** Concentrations of LPS, ALT, GGT, LDH and CK among Con, H1N1 and H3N2 groups. Data was presented as the mean± SEM and analyzed by unpaired two-tailed t test. *P< 0.05, **P< 0.01, ***P< 0.001, compared with Con group; #*P*< 0.05, compared with H1N1 group.

The concentrations of total protein (TP) and albumin (ALB) were significantly increased after IAV infection, while was decreased in both H1N1 and H3N2 subgroups (**Figure 2A**). The concentration of triglyceride (TG) was decreased in patients with IAV infection, C3 complement (C3) and calcium (Ca) was significantly decreased in both H1N1 and H3N2 subgroups (**Figures 2B-D**). The content of direct bilirubin (DBIL) was decreased after both H1N1 and H3N2 infection, while the total bilirubin (TBIL) and indirect bilirubin (IBIL) were decreased after H1N1 infection (**Figures 2E, F**). The concentration of lipase (LPS) was increased and gamma-glutamyltransferase (GGT) was decreased after IAV infection, especially those with H1N1 infection, respectively (**Figures 2G, H**). However, children with H3N2 infection displayed lower concentrations of alanine transferase (ALT) and lactate dehydrogenase (LDH). In addition, children with H3N2 subtype had lower LDH and creatine kinase (CK) contents than those with H1N1 infection (**Figures 2G, H**). After IAV infection, boys had lower C3, C4, sCRP and higher LPS than girls (**Supplementary Figures S2A-C**). Compared with BPI group, the CREA was increased in UI group, which was absent from IAV infection (**Supplementary Figure S2D**). The concentrations of PA, GB, A:G, AST, ALP, CHE, UREA, UA, HBDH, Mg, TCHOL, CG, IgA, IgG, IgM in IAV patients were not statistically different from those of the control group (**Supplementary Tables S6, S7**).

### 3.3 IAV infection altered serum metabolic profile in pediatric patients

**Figure 3:**
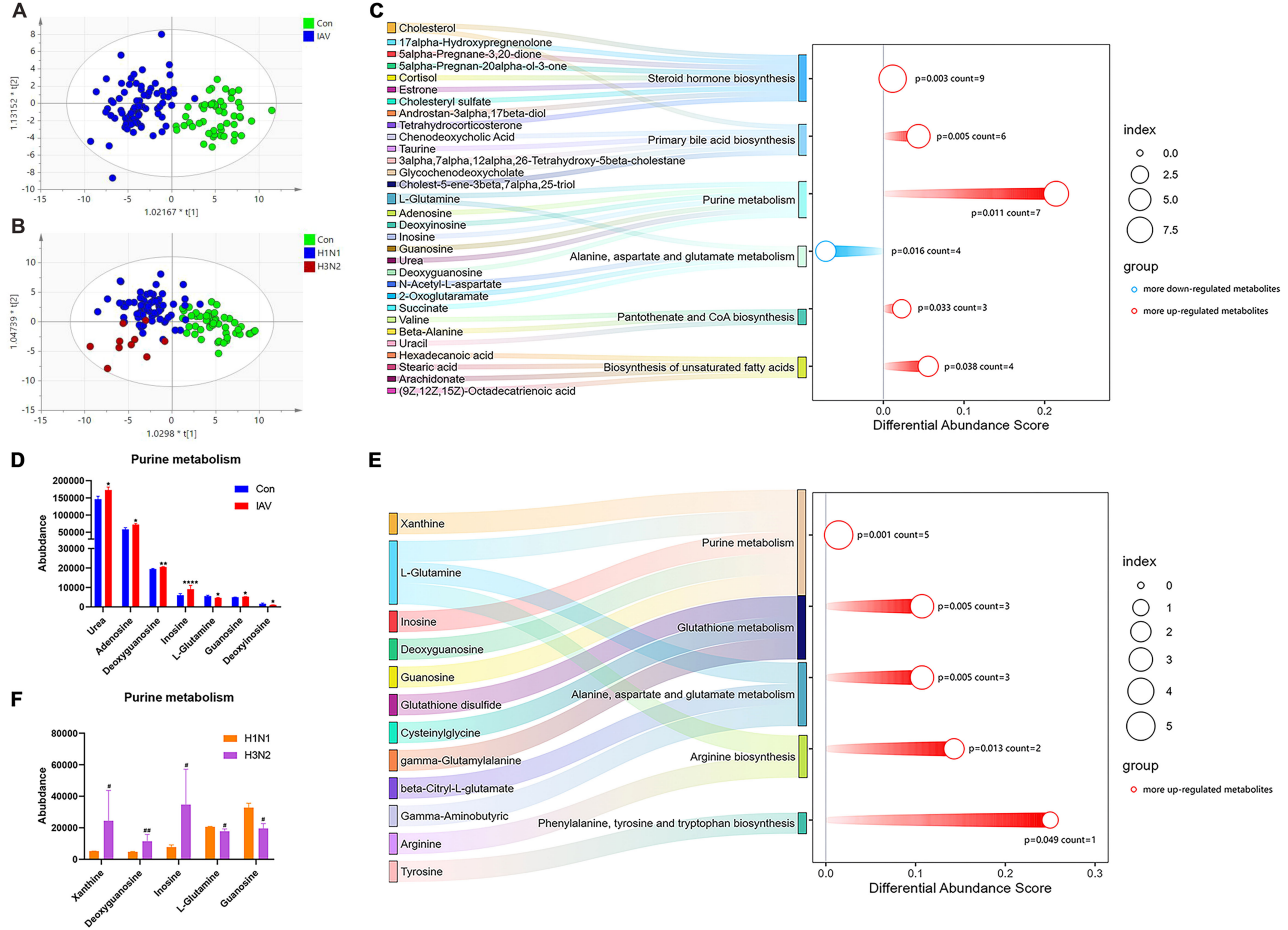
Serum metabolic profiling and pathway analysis. **(A)** The OPLS-DA plot of the serum samples from Con and IAV groups, including **(B)** H1N1 and H3N2 groups. **(C)** Pathway analysis of differential metabolites between the Con and IAV groups or **(E)** H1N1 and H3N2 groups. **(D)** The relative intensity of metabolites involved in the purine metabolism pathway between Con and InfA groups or **(F)** H1N1 and H3N2 groups. Data was presented as the mean± SEM and analyzed by unpaired two-tailed t test. *P< 0.05, **P< 0.01, ****P<0.0001, compared with Con group; #*P*< 0.05, ##*P*<0.01, compared with H1N1 group.

Principal components analysis (PCA) of serum metabolomics showed good clustering of QC samples, indicating high data stability (Fig.S3A). The Orthogonal Partial Least Squares-Discriminant Analysis (OPLS-DA) plots revealed different metabolic profile between control and IAV groups (**Figure 3A**). There were 209 differential metabolites identified between control and IAV groups. Pediatric patients infected by H1N1 and H3N2 also had different serum metabolic profiles (**Figure 3B**), and 37 differential metabolites were observed. IAV infection significantly altered 6 pathways, including steroid hormone biosynthesis, primary bile acid biosynthesis, purine metabolism, alanine, aspartate and glutamate metabolism, pantothenate and COA biosynthesis and biosynthesis of unsaturated fatty acids (**Figure 3C**). Urea, adenosine, deoxyguanosine, inosine and guanosine were outlier upregulated metabolites in IAV-infected patients (**Figure 3D**). Valine, leucine and isoleucine biosynthesis was the only overlapping pathway enriched in both H1N1 and H3N2 infection (**Supplementary Figures S3B, C**). Compared with H1N1-infected patients, purine metabolism was a key pathway altered in H3N2-infected patients (**Figure 3E**). Moreover, serum concentrations of xanthine, deoxyguanosine, inosine were significantly upregulated by H3N2 infection in comparison with H1N1 infection (**Figure 3F**).

### 3.4 Influenza A virus altered the composition and predictive function of respiratory microbiota

**Figure 4:**
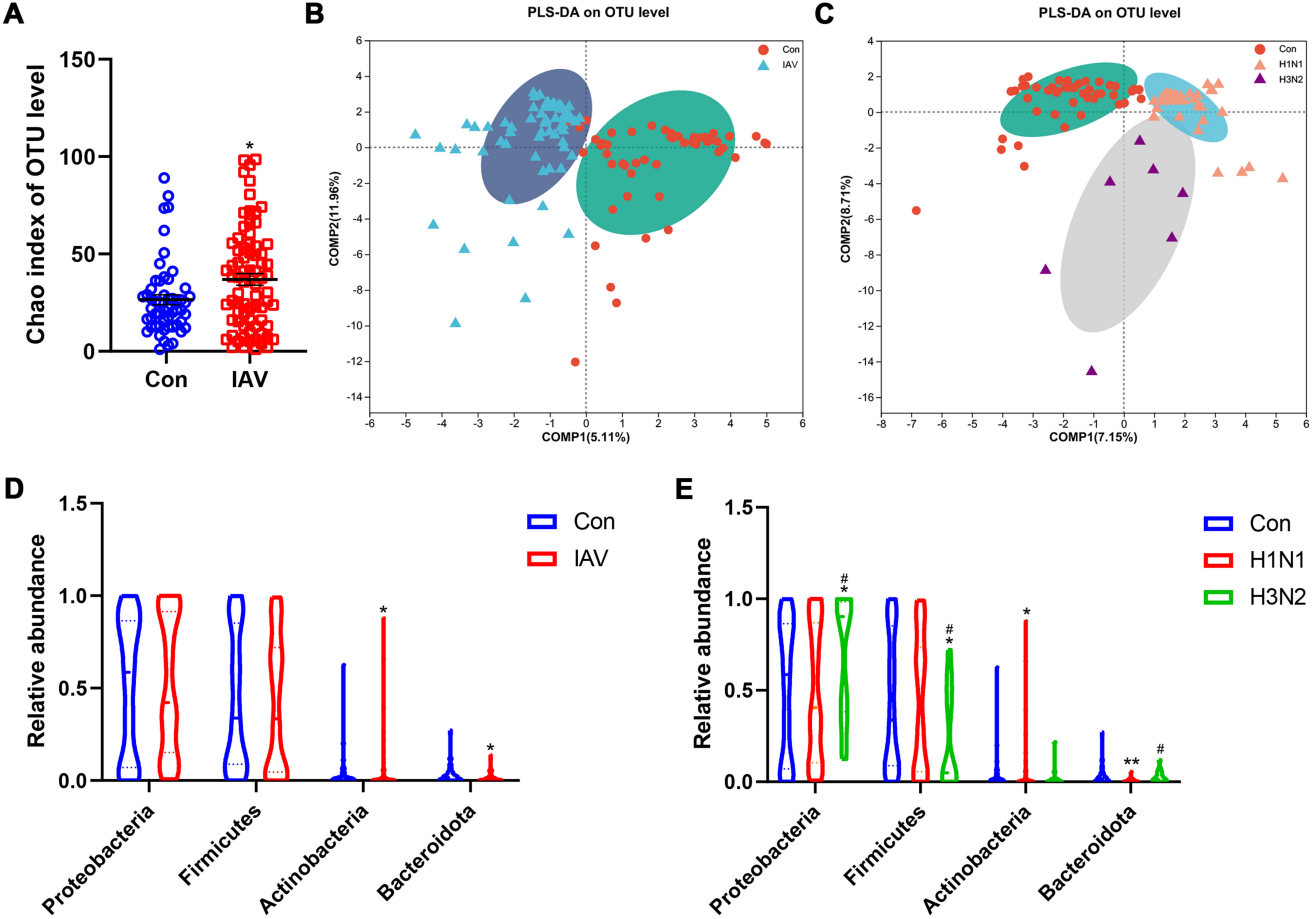
Effects IAV infection on the microbiota from upper respiratory tract. **(A)** Alpha diversity analysis of Chao index between the Con and IAV groups at the OTU level. **(B)** Beta diversity analysis using the projection to latent structures discriminant analysis (PLS-DA) based on binary-Lennon analysis (beta diversity) on the OTU among Con and IAV groups or **(C)** H1N1 and H3N2 groups. **(D)** Relative abundance on the phylum level among control and IAV groups or **(E)** H1N1 and H3N2 groups. Data was presented as the mean± SEM and analyzed by Wilcoxon rank-sum test. *P< 0.05, **P< 0.01, compared with Con group; #*P*< 0.05, compared with H1N1 group.

16S rRNA sequencing analysis of sputum and nasal lavage fluid was performed to determine the potential changes in the respiratory microbiome caused by IAV. Compared with the control group, the increase of Chao index in IAV group indicated an increase in microbial richness (**Figure 4A**; **Supplementary Figures S4A, B**). PLS-DA analysis showed that the structure of microbiota from URT and LRT of the control group was different from that of IAV patients, including H1N1 and H3N2 (**Figures 4B, C**; **Supplementary Figures S4C, D**). However, there were group differences in the proportion of bacterial communities represented by these dominant phylum. The proportion of *Bacteroidetes* in the IAV group was smaller than that in the control group, but the amount of *Actinobacteria* in the IAV group was higher than that in the control group (**Figure 4D**). The H3N2 subtype was more prevalent in *Proteobacteria* and fewer in *Firmicutes*. Compared with the control group, the amount of *Actinobacteria* was increased and the amount of *Bacteroidetes* was decreased in the H1N1 group (**Figure 4E**). In the URT, comparisons between groups showed multiple genus-level differences in bacterial taxa. Compared with the Con group, the IAV group had significantly higher abundance of *Moraxella* and *Haemophilus*. When comparing the two influenza subtypes, the abundance of *Ureaplasma* was higher in H3N2 group than in the H1N1 group. In the LRT, an increased abundance of Moraxella was also observed (**Supplementary Figures S4D-H**).

PICRUST2.0 was used to predict the functional characteristics of respiratory microbiota. In the URT, 180 metabolic pathways were identified between the control group and the IAV group, characterized by purine metabolism (**Supplementary Figure S5A**). There 175 and 58 metabolic pathways were identified in H1N1 and H3N2 group, respectively (**Supplementary Figures S5B, C**). 40 overlapped pathways were found between H1N1 and H3N2 group (**Supplementary Figure S5D**). Compared with H1N1 group, 14 pathways were identified in H3N2 group (**Supplementary Figure S5E**). There 34 metabolic pathways were significantly disrupted in the LRT (**Supplementary Figure S5F**).

### 3.5 Correlation between the microbiota community structure and differential metabolites

**Figure 5:**
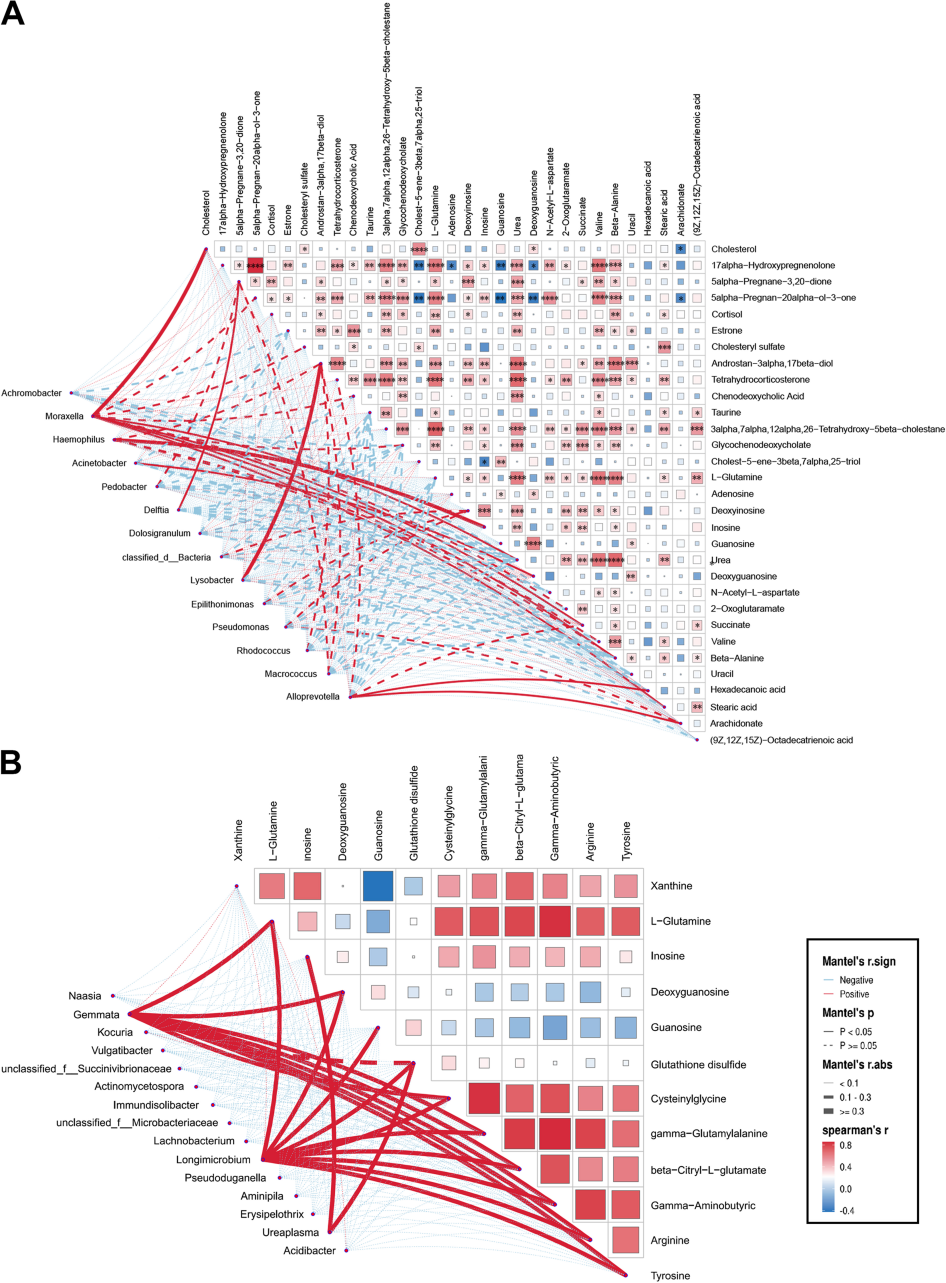
Association of discrepant genera correlate with metabolites in the upper respiratory tract. **(A)** Mantel test of differential genera with metabolites in differential metabolites between Con and IAV or **(B)** H1N1 and H3N2 groups. Significant correlations are denoted by stars (*P < 0.05, **P < 0.01, ***P < 0.001, ****P < 0.0001).

The Mantel test was performed to assess the relationship between different respiratory bacterial genera and their differential metabolites. *Moraxella* in the URT was positively correlated with several metabolites between the control and IAV groups. In addition, the relative abundance of *Haemophilus* was proportional to inosine respectively (**Figure 5A**). *Ureaplasma* in the URT was found to be proportional to inosine between H1N1 and H3N2 groups (**Figure 5B**). *Moraxella*, *Acinetobacter* and *Haemophilus* in the URT were not associated with any differential metabolites between control and H1N1 groups (**Supplementary Figure S6A**). The relative abundance of *Acinetobacter* was proportional to glutathione disulfide, beta-citryl-L-glutamate, 3-carboxy-1-hydroxypropyl-thPP between the control and H3N2. *Moraxella* was proportional to citric acid, beta-citryl-L-glutamate (**Supplementary Figure S6B**). Only *Moraxella* in LRT was associated with serum differential metabolites between the control and IAV groups (**Supplementary Figure S6C**).

## 4 Discussion

Influenza virus stands as one of the leading causes of acute respiratory illness among children. Currently, IAV infection (H1N1) is widespread in humans, often resulting in more severe symptoms (Gaitonde et al., 2019). Viruses have evolved mechanisms to evade host immune responses, including antigen transfer to evade vaccine protection. Moreover, commercially available anti-influenza drugs face limitations in terms of administration time and drug resistance. Therefore, there is an urgent need to develop drugs targeting metabolic mechanisms (Zumla et al., 2016). The oropharynx plays a pivotal role as a bridge between the external environment and the respiratory system. The advancement in technology has increasingly focused attention on the interaction of respiratory microbiome and metabolome in the context of IAV infection (Hu et al., 2022). Hence, this study aimed to characterize the serum metabolome and respiratory microbiota in children with IAV infection, including H1N1 and H3N2.

Lymphocytes play a crucial role in adaptive immunity against IAV infection, characterized by antigen-specific memory cells that capture and neutralize pathogens (Chen et al., 2018). The counts of lymphocytes, CD3^+^T cells, CD4^+^ T cells, CD8^+^ T cells and B cells in peripheral blood of IAV group were decreased. The reduction in T cells is in line with a diminished antibody response, as these cells play a crucial role in controlling influenza virus through cytokine production and support of B cell function (Bialka et al., 2024). Thrombocytopenia is a common occurrence in many viral infections. Both Middle East Respiratory Syndrome (MERS) and Severe Acute Respiratory Syndrome Coronavirus (SARS-CoV) infections have been associated with reduced Plt counts in peripheral blood (Chen et al., 2021; Jan et al., 2024). In our study, Plt was significantly lower in the IAV group, potentially due to disruption in megakaryocyte formation and reduced Plt cycle times, leading to decreased Plt counts. Additionally, the virus can produce molecules that promote Plt adhesion and aggregation, forming circulating complexes and exacerbating Plt decline (Bote et al., 2022). Serum TP and ALB was elevated in patients with IAV infection suggested that those proteins are key factors in viral replication and antiviral defense (Huang et al., 2024). Increased LPS could cause pancreatic damage, which may affect related immune cells (Sadeghi et al., 2020). Consistent with a retrospective cohort study, low serum bilirubin levels were observed in influenza patients found, our findings, emphasizing the importance of low bilirubin in acute viral or inflammatory disease (Sutton et al., 2024). Elevated AST and GGT levels are a common observation during liver injury or systemic infections (Zhang et al., 2019). In addition, elevated serum levels of LDH and CK are associated with influenza mortality (Yuan et al., 2020). However, lower levels of LDH and CK were observed in children with IAV infection. This inconsistency was result from unhealthy control and should be interpreted with caution. Studies have shown that CRP levels are significantly increased in children with severe influenza infection, which may be a potential indicator for identification and severity assessment of children with influenza (Zou et al., 2021). Women had an increased risk of influenza-associated in-hospital death (Karolyi et al., 2021). Thus, we compared the effect of gender differences on influenza in children. In line with previous study, higher levels of CRP, sCRP and LPS were observed in girls. Metabolomics approach had provided insightful evidence of altered metabolic profile and enriched metabolic pathways in IAV infection. A previous study revealed the disruption of purine metabolism in human airway epithelial cells infected with IAV (Chow et al., 2020). Here, untargeted metabolomics analysis revealed 6 disrupted metabolic pathways between the control and IAV group, including purine metabolism. Studies have shown that depletion of L-glutamine weakens humoral or cellular immunity following influenza virus infection (Pierre et al., 2016; Monteiro et al., 2020). Additionally, influenza polymerase has been shown to prime cRNA with guanosine, favoring the synthesis of transcribed virion RNA (Pagadala et al., 2020). Moreover, IAV infection has been found to increase adenosine production in mice, and activation of A1 subtype adenosine receptors by adenosine contributes to the development of acute lung injury after influenza virus infection (Simmons et al., 2024). Notably, serum inosine and urea were correspondingly increased in the IAV group, consistent with previous studies on altered metabolic levels in mice after IAV infection. The catabolic degradation of nucleic acids to these deamination products can serve as substrates for xanthine oxidase and contribute to superoxide production and influenza pathogenesis (Gutierrez et al., 2024). Compared with H1N1 group, the levels of xanthine, guanosine and inosine in H3N2 group infected group were increased, while the level of L-glutamine was decreased. Therefore, we speculate that these changes may aggravate H3N2- infection by disrupting humoral or cellular immunity (Pierre et al., 2016; Simmons et al., 2024). Although the mechanisms underlying the involvement of steroid hormone biosynthesis in influenza viruses remain unclear, corticosteroids are commonly used clinically to treat critical illness associated with IAV (Delaney et al., 2016).

In patients with severe pneumonia, whether positive and negative for influenza virus, Shannon’s index was slightly higher, although the difference was not statistically significant (Zhou et al., 2023). This is consistent with our findings, which showed that the diversity of respiratory microbiota in children infected with IAV was similar to that of the Con group, except for an increase in the richness of the flora. The URT microbiota of patients with H1N1 influenza virus are mainly composed of *Actinobacteria*, *Firmicutes* and *Proteobacteria* at the phylum level (Gu et al., 2023). *Moraxella and Haemophilus* were the dominant genera, and both belonged to *Proteobacteria*, which was increased after IAV infection. *Haemophilus* is considered harmful to children and is often found to be increased in patients with viral infections (Kloepfer and Kennedy, 2023). *Moraxella* species were reported to be elevated in patients with IAV viral infections (Thurner et al., 2020). The higher abundance of *Ureaplasma* in the H3N2 group may have considerable proinflammatory capacity in human monocytes compared with H1N1, which could promote the occurrence of the H3N2 virus (Viscardi et al., 2023).

Further comprehensive multi-omics association analysis revealed significant correlation between respiratory microbiota and serum metabolites. For instance, *Haemophilus* and *Ureaplasma* were both positively correlated with inosine. The addition of inosine for a certain period of time could stimulate protein synthesis and promote the growth of *Haemophilus in vitro (* Lichtenegger et al., 2014). In patients with influenza, changes in *Haemophilus* in the respiratory tract were significantly associated with influenza severity and with changes in metabolites such as inosine, xanthine, and hypoxanthine (Cui et al., 2017; Wen et al., 2018). Several studies have found a strong correlation between elevated *Haemophilus* levels and changes in purine metabolites. Previous studies on patients with chronic heart failure found that the purine metabolism was the most enriched pathway positively correlated with the abundance of *Haemophilus (* Wang et al., 2021). There are few reports on the relationship between inosine and *Ureaplasma*. The presence of inosine was only found to promote the growth of *Ureaplasma* through the formation of hypoxanthine (Bautista et al., 2013). These results indicated that patients infected with IAV exhibited changes in the exo-pharyngeal microbiota and its metabolites, with a notable significant correlation between the oropharyngeal microbiota and oropharyngeal metabolites (Euba et al., 2023; Teng et al., 2023). Combined with these findings, It is of great significance to study the pathogenesis of influenza in children through respiratory microbiota and purine metabolism. The utilization of multi-omics analysis revealed significant changes in respiratory microbiota and serum metabolites in patients with IAV, along with significant correlations between them. However, this study has several limitations. Firstly, these findings are based on data collected from a single hospital with a relatively small sample size. Thus, larger multicenter studies are needed to validate our metagenomics and metabolomics findings. Second, the analysis was restricted to pediatric patients with IAV infection and those who tested negative for 13 types of pathogens. Thus, the generalizability of our findings to healthy children and children with mixed viral infections remains uncertain. Future studies should include analyses of healthy children as well as other important respiratory viruses to allow a more comprehensive assessment of available molecular diagnostics. Lastly, although the selection of subjects adhered to rigorous criteria, ethical considerations for prompt medication administration to children may introduce various underlying factors affecting the microbiome and metabolome. Thus, there may still be various confounding factors affecting the microbiome and metabolome. More in-depth longitudinal study is needed to minimize confounding factors and dynamically track changes in the respiratory microbiome and metabolome of children with IAV infection.

## Data Availability

The original contributions presented in the study are included in the article/**Supplementary Material**. Further inquiries can be directed to the corresponding authors.
