# Supplementary material for: Characterization of serum metabolome and respiratory microbiota in children with influenza A virus infection
Source: Front Cell Infect Microbiol. 2025 Jan 30;14:1478876. doi: 10.3389/fcimb.2024.1478876 (PMC11821643; doi:10.3389/fcimb.2024.1478876)
Supplement: Supplementary file 1 [file DataSheet1.docx]

**Characterization of serum metabolome and respiratory microbiota in children with Influenza A virus infection**

Xinyi Shi^1ǂ^, Shenghao Hua^2ǂ^, Mengqing Xiao^3^, Wenlong Pei^1^, Zeyuan Chen^1^, Zhe Cao^4^, Zhan Zhang^1^, Xuejun Shao^2^*, Yu Xia^5,6^*

**Supplementary data**

**Figure legend**

**Figure S1. The effects of IAV infection on blood cells in patients with different gender and clinical symptoms.** (A) Serum content of basophil (BA), platelet (Plt) and CD8^+^ T cell in patients with bronchopneumonia (BPI) or upper respiratory tract infection (UI). and GI and BI groups. The percentage of (B) plateletcrit (Pct), (C) CD3^+^ T cells, (D) CD4^+^ T cell, (E) NK cells. (F) The concentration of C-reactive protein (CRP) in boys (BI) and girls (GI) with IAV infection. Data was presented as the mean± SEM and analyzed by unpaired two-tailed t test. **P*< 0.05, compared with the respective group.

**Figure S2. The effects of IAV infection on serum chemistry in patients with different gender and clinical symptoms. (A)** Concentrations of complement 3 (C3) and C4, (B) hypersensitive C-reactive protein (sCRP), and (C) lipase (LPS) between GI and BI groups. (D) The concentration of creatinine in patients with bronchopneumonia (BPI) or upper respiratory tract infection (UI). Data was presented as the mean± SEM and analyzed by unpaired two-tailed t test. **P*< 0.05, ***P*< 0.01, compared with the respective group.

**Figure S3. Serum metabolic profiling and pathway analysis.** Pathway analysis of differential metabolites between the Con and H1N1 or (B) H3N2 groups.

**Figure S4. The composition of respiratory microbiota after influenza A virus infection**. (A) Shannon and (B) Chao indices of the two groups at the OTU level on the lower respiratory tract (LRT). (C) PLS-DA analysis of LRT microbiota on the OTU between the control and IVA groups. (D) Taxonomic differences of upper respiratory tract (URT) microbiota between Con and IAV groups or (E) H1N1 and H3N2 groups, (F) Con and H1N1, (G) Con and H3N2 groups at the genus level. (H) Taxonomic differences of LRT microbiota between Con and IAV groups. Differences between groups were determined using the Wilcoxon rank-sum test.

**Figure S5. Predicted functions of the metagenome from 16S rRNA sequencing.** Functional annotation of the predicted metagenome based on 16S rRNA data was performed by PICRUSt and STAMP was used to identify significant pathways. (A) Enriched pathways of URT microbiota between Con and IAV groups. (B) Enriched pathways of URT microbiota between Con and H1N1 groups. (C) Enriched pathways of URT microbiota between Con and H3N1 groups. (C) Enriched pathways between H1N1 and H3N2 groups on the upper respiratory tract. (D) The common enriched pathways of URT microbiota between H1N1 and H3N2 groups in comparison with the control group. (E) Enriched pathways of URT microbiota between H1N1 and H3N2 groups. (F) Enriched pathways of LRT microbiota between Con and IAV groups. Differences between groups were determined using the Welsh’s t-test.

**Figure S6. Association of differential genera with differential metabolites.** (A) Mantel test of differential genera from URT with metabolites in differential metabolites after H1N1 (B) H3N2 infection in comparison with the control group. (C) Mantel test of differential genera from LRT with metabolites in differential metabolites between Con and IAV. Significant correlations are denoted by stars (**P* < 0.05, ***P* < 0.01, ****P* < 0.001).

**Table S1. Baseline sociodemographics and clinical characteristics.**

| **Characteristics** | **Con** | **IAV** | ***P* value** |
| --- | --- | --- | --- |
|  | **n (%)** | **n (%)** |  |
| **Age(year)** | 4.29±4.22 | 4.85±2.46 | 0.325 |
| **Gender** |  |  | 0.321 |
| Female | 28 (50.9) | 36 (42.4) |  |
| Male | 27 (49.1) | 49 (57.6) |  |
| **Virus subtypes** |  |  |  |
| H1N1 |  | 68 (80) |  |
| H3N2 |  | 17 (20) |  |
| **Clinical symptoms** |  |  |  |
| Bronchopneumonia | 26 (47.3) | 43 (50.6) | 0.732 |
| Bronchitis | 5 (9.1) | 5 (5.9) | 0.514 |
| Upper respiratory tract infection (URTI) | 12 (21.8) | 28 (32.9) | 0.183 |
| Others | 12 (21.8) | 9 (10.6) | 0.09 |

**Table S2. Comparison of blood routine test between Con and InfA groups.**

|  | Con (n=44) | IAV (n=55) | *P* value |
| --- | --- | --- | --- |
| RBC (10^12^/L) | 4.28±0.62 | 4.54±0.37 | 0.011 |
| WBC (10^9^/L) | 9.21±4.03 | 6.77±3.26 | 0.0012 |
| NE (10^9^/L) | 4.50±3.29 | 3.81±2.98 | 0.276 |
| LY (10^9^/L) | 3.80±2.28 | 2.36±1.53 | 0.0003 |
| MO (10^9^/L) | 0.67±0.38 | 0.53±0.35 | 0.068 |
| EO (10^9^/L) | 0.21±0.29 | 0.05±0.06 | <0.0001 |
| BA (10^9^/L) | 0.03±0.03 | 0.01±0.01 | 0.0003 |
| Plt (10^9^/L) | 351.48±139.64 | 254.47±110.01 | 0.0002 |
| NE (%) | 46.97±20.76 | 51.89±23.88 | 0.283 |
| LY (%) | 42.99±19.37 | 38.74±22.74 | 0.326 |
| MO (%) | 7.48±2.94 | 8.34±4.41 | 0.273 |
| EO (%) | 2.28±2.87 | 0.84±1.16 | 0.001 |
| BA (%) | 0.28±0.25 | 0.17±0.15 | 0.011 |
| Pct (%) | 0.33±0.12 | 0.24±0.10 | 0.0001 |
| Hgb (g/L) | 116.45±14.28 | 118.95±8.34 | 0.281 |
| Hct (L/L) | 0.35±0.04 | 0.36±0.03 | 0.08 |
| MCV (fL) | 82.27±6.30 | 80.11±5.32 | 0.067 |
| MCH (pg) | 27.60±4.35 | 26.31±2.01 | 0.054 |
| MCHC (g/L) | 334.63±38.61 | 328.45±14.31 | 0.275 |
| RDW (%) | 13.29±1.63 | 13.21±0.79 | 0.748 |
| MPV (fL) | 9.73±1.07 | 9.60±1.06 | 0.538 |
| PDW (%) | 12.78±2.90 | 13.53±2.65 | 0.182 |
| CRP (mg/L) | 8.56±14.42 | 6.27±14.80 | 0.45 |

RBC: red blood cell; WBC: white blood cell; NE: neutrophil; LY: lymphocyte; EO: eosinophil; BA: basophil; Plt: platelet; Hgb: Hemoglobin; Hct: hematocrit; MCV: mean corpuscular volume; MCH: mean corpuscular hemoglobin; MCHC: mean corpuscular hemoglobin concentration; RDW: red blood cell distribution width; Pct: plateletcrit; MPV: mean platelet volume; PDW: platelet distribution width; CRP: C-reactive protein.

**Table S3. Comparison of blood routine test** **among Con, H1N1 and H3N2 groups.**

|  | Con  (n=44) | H1N1  (n=47) | H3N2  (n=8) | H1N1 vs Con | H3N2 vs Con | H1N1 vs H3N2 |
| --- | --- | --- | --- | --- | --- | --- |
| RBC (10^12^/L) | 4.28±0.62 | 4.55±0.39 | 4.50±0.28 | 0.052 | 0.299 | 0.972 |
| WBC (10^9^/L) | 9.21±4.03 | 9.94±3.34 | 5.75±2.72 | 0.014 | 0.026 | 0.619 |
| NE (10^9^/L) | 4.50±3.29 | 3.95±3.10 | 2.99±2.07 | 0.797 | 0.277 | 0.61 |
| LY (10^9^/L) | 3.80±2.28 | 2.41±1.58 | 2.10±1.19 | 0.004 | 0.017 | 0.88 |
| MO (10^9^/L) | 0.67±0.38 | 0.52±0.37 | 0.60±0.20 | 0.182 | 0.832 | 0.758 |
| EO (10^9^/L) | 0.21±0.29 | 0.05±0.06 | 0.05±0.06 | 0.002 | 0.003 | 0.998 |
| BA (10^9^/L) | 0.03±0.03 | 0.01±0.01 | 0.02±0.02 | 0.0002 | 0.72 | 0.037 |
| PLT (10^9^/L) | 351.48±139.64 | 263.55±114.81 | 201.13±54.13 | 0.005 | 0.001 | 0.067 |
| NE (%) | 46.97±20.76 | 52.45±24.74 | 48.61±19.04 | 0.582 | 0.994 | 0.941 |
| LY (%) | 42.99±19.37 | 38.77±23.59 | 38.53±18.21 | 0.726 | 0.892 | 1 |
| MO (%) | 7.48±2.94 | 7.77±4.26 | 11.66±4.02 | 0.974 | 0.06 | 0.086 |
| EO (%) | 2.28±2.87 | 0.84±1.18 | 0.85±1.07 | 0.009 | 0.054 | 1 |
| BA (%) | 0.28±0.25 | 0.16±0.14 | 0.35±0.35 | 0.025 | 0.913 | 0.401 |
| PCT (%) | 0.33±0.12 | 0.25±0.11 | 0.21±0.06 | 0.002 | 0.001 | 0.47 |
| Hgb (g/L) | 116.45±14.28 | 118.53±8.75 | 121.38±5.13 | 0.791 | 0.243 | 0.507 |
| Hct (L/L) | 0.35±0.04 | 0.36±0.27 | 0.37±0.03 | 0.343 | 0.322 | 0.887 |
| MCV (fL) | 82.27±6.30 | 79.83±5.60 | 81.75±2.96 | 0.153 | 0.975 | 0.408 |
| MCH (pg) | 27.60±4.35 | 26.20±2.14 | 27.00±0.82 | 0.161 | 0.784 | 0.191 |
| MCHC (g/L) | 334.63±38.61 | 328.11±14.67 | 330.50±12.63 | 0.648 | 0.921 | 0.947 |
| RDW (%) | 13.29±1.63 | 13.26±0.80 | 12.95±0.66 | 0.999 | 0.677 | 0.578 |
| MPV (fL) | 9.73±1.07 | 9.41±0.97 | 10.69±0.89 | 0.371 | 0.058 | 0.052 |
| PDW (%) | 12.78±2.90 | 13.51±2.67 | 13.69±2.66 | 0.521 | 0.766 | 0.997 |
| CRP (mg/L) | 8.56±14.42 | 6.41±15.89 | 5.54±6.61 | 0.881 | 0.724 | 0.99 |

RBC: red blood cell; WBC: white blood cell; NE: neutrophil; LY: lymphocyte; MO: monocyte; EO: eosinophil; BA: basophil; Plt: platelet; Hgb: hemoglobin; Hct: hematocrit; MCV: mean corpuscular volume; MCH: mean corpuscular hemoglobin; MCHC: mean corpuscular hemoglobin concentration; RDW: red blood cell distribution width; Pct: plateletcrit; MPV: mean platelet volume; PDW: platelet distribution width; CRP: C-reactive protein.

**Table S3. Comparison of CD indexes between Con and InfA groups.**

|  | Con (n=32) | IAV (n=54) | *P* value |
| --- | --- | --- | --- |
| lymphocyte (10^9^/L) | 4140.53±3007.90 | 2365.69±1838.51 | 0.001 |
| CD3^+^ (10^9^/L) | 2933.34±2296.35 | 1537.05±1199.58 | 0.001 |
| CD3^+^CD4^+^ (10^9^/L) | 1643.21±1210.58 | 862.63±744.43 | 0.001 |
| CD3^+^CD8^+^ (10^9^/L) | 1061.48±1108.54 | 526.90±400.44 | 0.002 |
| CD3^-^CD19^+^ (10^9^/L) | 738.46±596.13 | 500.71±498.05 | 0.049 |
| NK (10^9^/L) | 432.93±347.33 | 308.08±308.07 | 0.087 |
| CD3^+^ (%) | 69.05±10.17 | 64.93±9.84 | 0.067 |
| CD3^+^CD4^+^ (%) | 39.23±9.73 | 34.75±8.56 | 0.028 |
| CD3^+^CD8^+^ (%) | 24.31±8.31 | 23.02±6.41 | 0.042 |
| CD4^+^CD8^+^ (%) | 1.88±0.97 | 1.64±0.68 | 0.188 |
| CD3^-^CD19^+^ (%) | 18.27±7.32 | 20.37±8.30 | 0.238 |
| CD3^-^CD(16+56)^+^ (%) | 11.75±6.78 | 13.85±9.28 | 0.268 |
| CD19^+^CD23^+^ (%) | 6.70±3.14 | 5.97±3.40 | 0.328 |

**Table S4. Comparison of CD indexes among Con, H1N1 and H3N2 groups.**

|  | Con (n=32) | | H1N1 (n=41) | H3N2 (n=13) | H1N1 vs Con | H3N2 vs Con | H1N1 vs H3N2 |
| --- | --- | --- | --- | --- | --- | --- | --- |
| lymphocyte (10^9^/L) | | 4140.53±3007.90 | 2464.22±1956.12 | 2054.92±1427.03 | 0.025 | 0.009 | 0.798 |
| CD3^+^ (10^9^/L) | | 2933.34±2296.35 | 1599.74±1286.85 | 13339.33±833.11 | 0.015 | 0.005 | 0.796 |
| CD3^+^CD4^+^ (10^9^/L) | | 1643.21±1210.58 | 906.04±807.60 | 725.69±496.84 | 0.014 | 0.02 | 0.707 |
| CD3^+^CD8^+^ (10^9^/L) | | 1061.48±1108.54 | 544.79±425.89 | 470.46±314.76 | 0.050 | 0.026 | 0.873 |
| CD3^-^CD19^+^ (10^9^/L) | | 738.46±596.13 | 512.61±516.10 | 463.17±453.54 | 0.253 | 0.275 | 0.982 |
| NK (10^9^/L) | | 432.93±347.33 | 330.12±333.13 | 238.55±205.82 | 0.496 | 0.075 | 0.561 |
| CD3^+^ (%) | | 69.05±10.17 | 64.44±9.97 | 66.48±9.61 | 0.159 | 0.809 | 0.88 |
| CD3^+^CD4^+^ (%) | | 39.23±9.73 | 34.42±9.36 | 35.79±5.48 | 0.106 | 0.363 | 0.886 |
| CD3^+^CD8^+^ (%) | | 24.31±8.31 | 22.96±6.69 | 23.20±5.67 | 0.836 | 0.938 | 0.999 |
| CD4^+^CD8^+^ (%) | | 1.88±0.97 | 1.64±0.75 | 1.63±0.44 | 0.574 | 0.594 | 1 |
| CD3^-^CD19^+^ (%) | | 18.27±7.32 | 19.97±8.49 | 1.63±0.44 | 0.735 | 0.474 | 0.885 |
| CD3^-^CD(16+56)^+^ (%) | | 11.75±6.78 | 14.69±10.16 | 11.19±5.10 | 0.368 | 0.986 | 0.283 |
| CD19^+^CD23^+^ (%) | | 6.70±3.14 | 5.77±3.27 | 6.62±3.84 | 0.524 | 1 | 0.851 |

**Table S5. Comparison of serum biochemistry between Con and InfA groups.**

|  | Con (n=56) | IAV (n=83) | *P* value |
| --- | --- | --- | --- |
| TP (g/L) | 65.28±6.62 | 67.22±3.73 | 0.03 |
| PA (mg/L) | 136.95±41.59 | 135.16±31.96 | 0.775 |
| ALB (g/L) | 42.43±3.43 | 43.48±2.31 | 0.032 |
| GLB (g/L) | 23.09±4.75 | 23.74±2.97 | 0.324 |
| A:G | 1.92±0.43 | 1.86±0.26 | 0.339 |
| ALT (U/L) | 21.99±14.32 | 19.38±12.40 | 0.27 |
| AST (U/L) | 44.39±30.03 | 45.70±18.92 | 0.756 |
| ALP (U/L) | 191.46±67.92 | 184.65±51.95 | 0.512 |
| GGT (U/L) | 15.57±11.37 | 10.19±4.43 | 0.0002 |
| CHE (U/L) | 7517.68±1473.72 | 7964.12±1474.4 | 0.82 |
| TBIL (umol/L) | 9.35±7.30 | 5.94±2.70 | 0.0002 |
| DBIL (umol/L) | 3.91±4.56 | 2.17±0.94 | 0.001 |
| IBIL (umol/L) | 5.04±3.38 | 3.77±1.83 | 0.006 |
| UREA (mmol/L) | 3.54±1.13 | 3.63±1.07 | 0.658 |
| CREA (umol/L) | 28.98±11.26 | 30.92±7.67 | 0.23 |
| UA (umol/L) | 251.77±91.15 | 258.29±83.40 | 0.664 |
| LDH (U/L) | 343.69±98.20 | 337.19±92.63 | 0.694 |
| HBDH (U/L) | 268.62±79.05 | 270.93±75.10 | 0.863 |
| CK (U/L) | 113.81±61.92 | 130.32±73.36 | 0.173 |
| Ca (mmol/L) | 2.38±0.16 | 2.30±0.13 | 0.001 |
| Mg (mmol/L) | 1.04±0.10 | 1.01±0.10 | 0.124 |
| LPS (U/L) | 19.45±8.82 | 24.82±13.30 | 0.009 |
| TCHOL (mmol/L) | 3.77±0.79 | 3.86±0.63 | 0.46 |
| TG (mmol/L) | 1.10±0.65 | 0.88±0.49 | 0.026 |
| CG (ug/mL) | 5.37±5.48 | 4.23±2.95 | 0.118 |
| sCRP (mg/L) | 12.95±20.49 | 7.91±13.33 | 0.085 |
| C3 (g/L) | 1.12±0.31 | 0.97±0.16 | 0.0005 |
| C4 (g/L) | 0.31±0.12 | 0.32±0.09 | 0.553 |
| IgA (g/L) | 0.97±0.83 | 0.92±0.60 | 0.669 |
| IgG (g/L) | 6.97±2.66 | 7.48±2.10 | 0.23 |
| IgM (g/L) | 1.05±0.60 | 1.09±0.40 | 0.639 |

TP: total protein; PA: prealbumin; ALB: albumin; GLB: globulin; A:G: albumin: globulin; ALT: alanine transferase; AST: aspartate transaminase; ALP: alkaline phosphatase; GGT: gamma-glutamyltransferase; CHE: choline esterase; TBIL: total bilirubin; DBIL: direct bilirubin; IBIL: indirect bilirubin; CREA: creatinine; UA: uric acid; LDH: lactate dehydrogenase; HBDH: hydroxybutyrate dehydrogenase; CK: creatine kinase; Ca: calcium; Mg: magnesium; LPS: lipase; TCHOL: total cholesterol; TG: triglyceride; CG: cholyglycine; sCRP: hypersensitive C-reactive protein; C3: complement 3; C4: complement 4; IgA: immunoglobulin A; IgG: immunoglobulin G; IgM: immunoglobulin M.

**Table S6. Comparison of serum biochemistry among Con, H1N1 and H3N2 groups.**

|  | Con (n=56) | H1N1 (n=66) | H3N2 (n=17) | H1N1 vs Con | H3N2 vs Con | H1N1 vs H3N2 |
| --- | --- | --- | --- | --- | --- | --- |
| TP (g/L) | 65.52±6.80 | 67.22±3.72 | 67.21±3.90 | 0.263 | 0.489 | 1 |
| PA (mg/L) | 136.95±41.59 | 136.88±33.30 | 128.47±25.93 | 1.00 | 0.677 | 0.605 |
| ALB (g/L) | 42.43±3.43 | 43.55±2.37 | 43.21±2.12 | 0.118 | 0.589 | 0.918 |
| GLB (g/L) | 23.09±4.75 | 23.67±2.97 | 24.00±3.03 | 0.728 | 0.812 | 0.97 |
| A:G | 1.92±0.43 | 1.87±0.26 | 1.83±0.25 | 0.638 | 0.851 | 0.906 |
| ALT (U/L) | 21.99±14.32 | 20.52±13.27 | 15.09±7.16 | 0.921 | 0.035 | 0.081 |
| AST (U/L) | 44.39±30.03 | 47.45±19.98 | 39.15±12.66 | 0.671 | 0.893 | 0.12 |
| ALP (U/L) | 191.46±67.92 | 184.11±51.87 | 186.76±53.82 | 0.888 | 0. 988 | 0.997 |
| GGT (U/L) | 15.57±11.37 | 9.92±3.42 | 11.21±7.15 | 0.004 | 0.197 | 0.851 |
| CHE (U/L) | 7517.68±1473.72 | 7937.08±1415.08 | 8069.12±1729.61 | 0.302 | 0.561 | 0.988 |
| TBIL (umol/L) | 9.35±7.30 | 5.79±2.36 | 6.50±3.76 | 0.004 | 0.115 | 0.842 |
| DBIL (umol/L) | 3.91±4.56 | 2.14±0.88 | 2.28±1.16 | 0.02 | 0.059 | 0.948 |
| IBIL (umol/L) | 5.04±3.38 | 3.66±1.58 | 4.22±2.61 | 0.027 | 0.657 | 0.782 |
| UREA (mmol/L) | 3.54±1.13 | 3.59±1.11 | 3.75±0.91 | 0.991 | 0.827 | 0.912 |
| CREA (umol/L) | 28.98±11.26 | 30.71±7.45 | 31.72±8.69 | 0.698 | 0.646 | 0.96 |
| UA (umol/L) | 251.77±91.15 | 258.40±85.52 | 257.90±77.03 | 0.967 | 0.99 | 1 |
| LDH (U/L) | 343.69±98.20 | 347.77±97.29 | 296.11±57.16 | 0.994 | 0.049 | 0.021 |
| HBDH (U/L) | 268.62±79.05 | 278.14±79.28 | 242.94±48.26 | 0.882 | 0.296 | 0.075 |
| CK (U/L) | 113.81±61.92 | 140.12±78.71 | 93.44±26.17 | 0.125 | 0.159 | 0.001 |
| Ca (mmol/L) | 2.38±0.16 | 2.31±0.13 | 2.26±0.10 | 0.03 | 0.002 | 0.295 |
| Mg (mmol/L) | 1.04±0.10 | 1.02±0.11 | 1.00±0.07 | 0.497 | 0.215 | 0.876 |
| LPS (U/L) | 19.45±8.82 | 24.45±13.54 | 26.29±12.60 | 0.047 | 0.136 | 0.934 |
| TCHOL (mmol/L) | 3.77±0.79 | 0.87±0.52 | 0.92±0.37 | 0.955 | 0.523 | 0.722 |
| TG (mmol/L) | 1.10±0.65 | 0.87±0.52 | 0.92±0.37 | 0.107 | 0.396 | 0.965 |
| CG (ug/mL) | 5.37±5.48 | 4.15±2.76 | 4.57±3.67 | 0.365 | 0.870 | 0.957 |
| sCRP (mg/L) | 12.95±20.49 | 8.00±14.19 | 7.45±9.66 | 0.360 | 0.371 | 0.998 |
| C3 (g/L) | 1.12±0.31 | 0.97±0.17 | 0.97±0.12 | 0.011 | 0.016 | 0.998 |
| C4 (g/L) | 0.31±0.12 | 0.31±0.08 | 0.34±0.14 | 0.986 | 0.796 | 0.856 |
| IgA (g/L) | 0.97±0.83 | 0.86±0.53 | 1.14±0.78 | 0.797 | 0.842 | 0.436 |
| IgG (g/L) | 6.97±2.66 | 7.36±1.97 | 7.94±2.53 | 0.781 | 0.459 | 0.757 |
| IgM (g/L) | 1.05±0.60 | 1.08±0.43 | 1.15±0.28 | 0.991 | 0.760 | 0.801 |

TP: total protein; PA: prealbumin; ALB: albumin; GLB: globulin; A:G: albumin: globulin; ALT: alanine aminotransferase; AST: aspartate aminotransferase; ALP: alkaline phosphatase; GGT: gamma-glutamyltransferase; CHE: choline esterase; TBIL: total bilirubin; DBIL: direct bilirubin; IBIL: indirect bilirubin; CREA: creatinine; UA: uric acid; LDH: lactate dehydrogenase; HBDH: hydroxybutyrate dehydrogenase; CK: creatine kinase; CA: calcium; MG: magnesium; LPS: lipase; TCHOL: total cholesterol; TG: triglyceride; CG: cholyglycine; sCRP: hypersensitive C-reactive protein; C3: complement 3; C4: complement 4; IgA: immunoglobulin A; IgG: immunoglobulin G; IgM: immunoglobulin M.
