# Supplementary figures and images for: Characterization of serum metabolome and respiratory microbiota in children with influenza A virus infection
Source: Front Cell Infect Microbiol. 2025 Jan 30;14:1478876. doi: 10.3389/fcimb.2024.1478876 (PMC11821643; doi:10.3389/fcimb.2024.1478876)

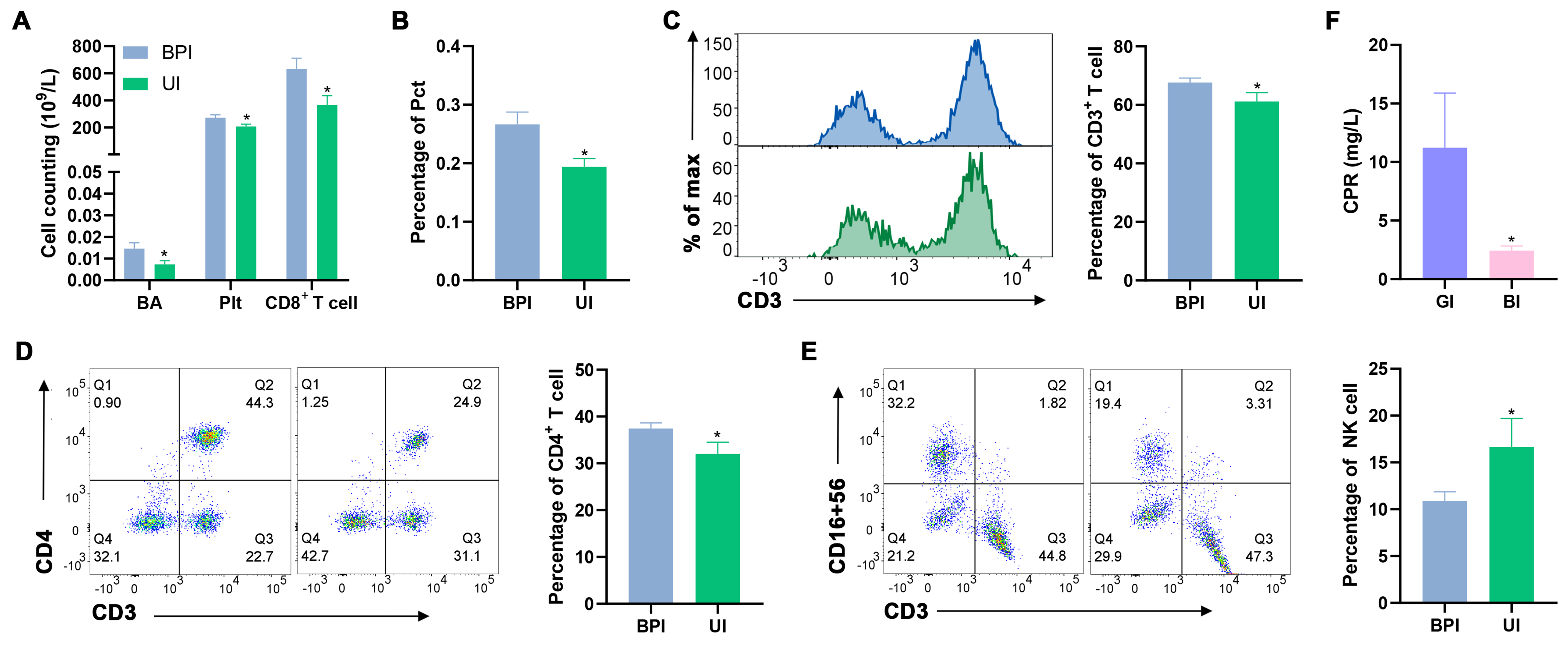

Supplement: Supplementary file 2 [file Image1.jpeg]

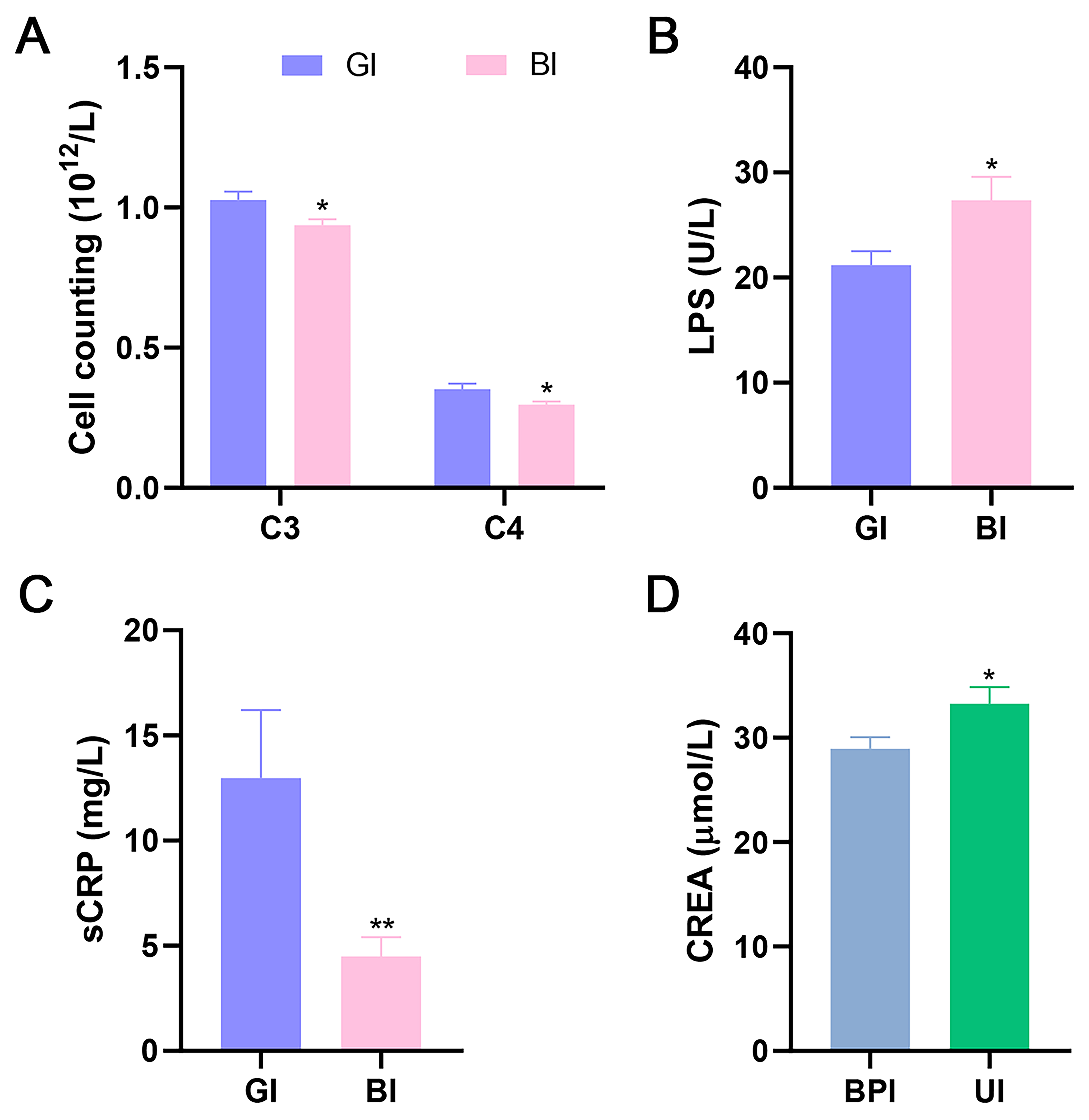

Supplement: Supplementary file 3 [file Image2.jpeg]

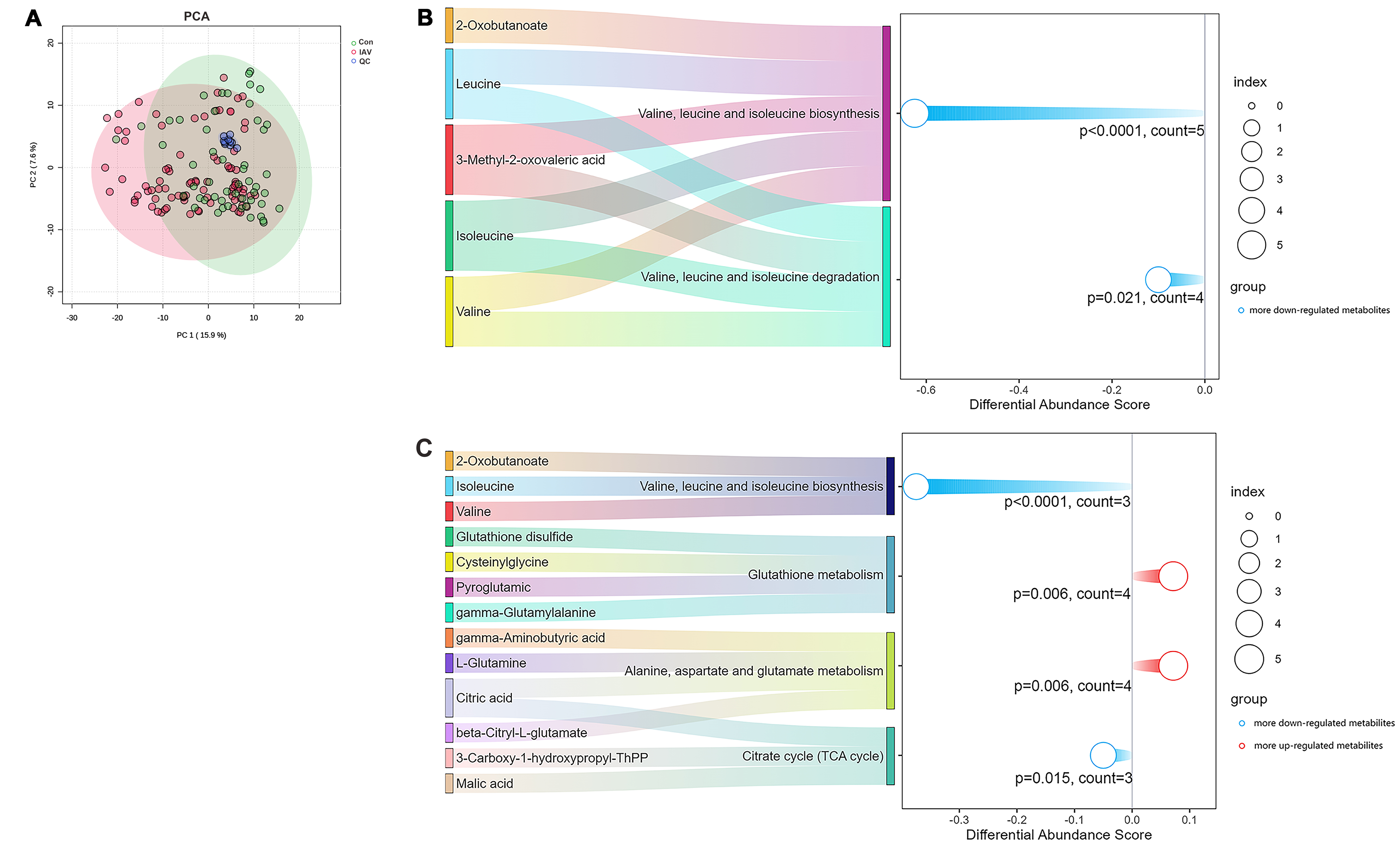

Supplement: Supplementary file 4 [file Image3.jpeg]

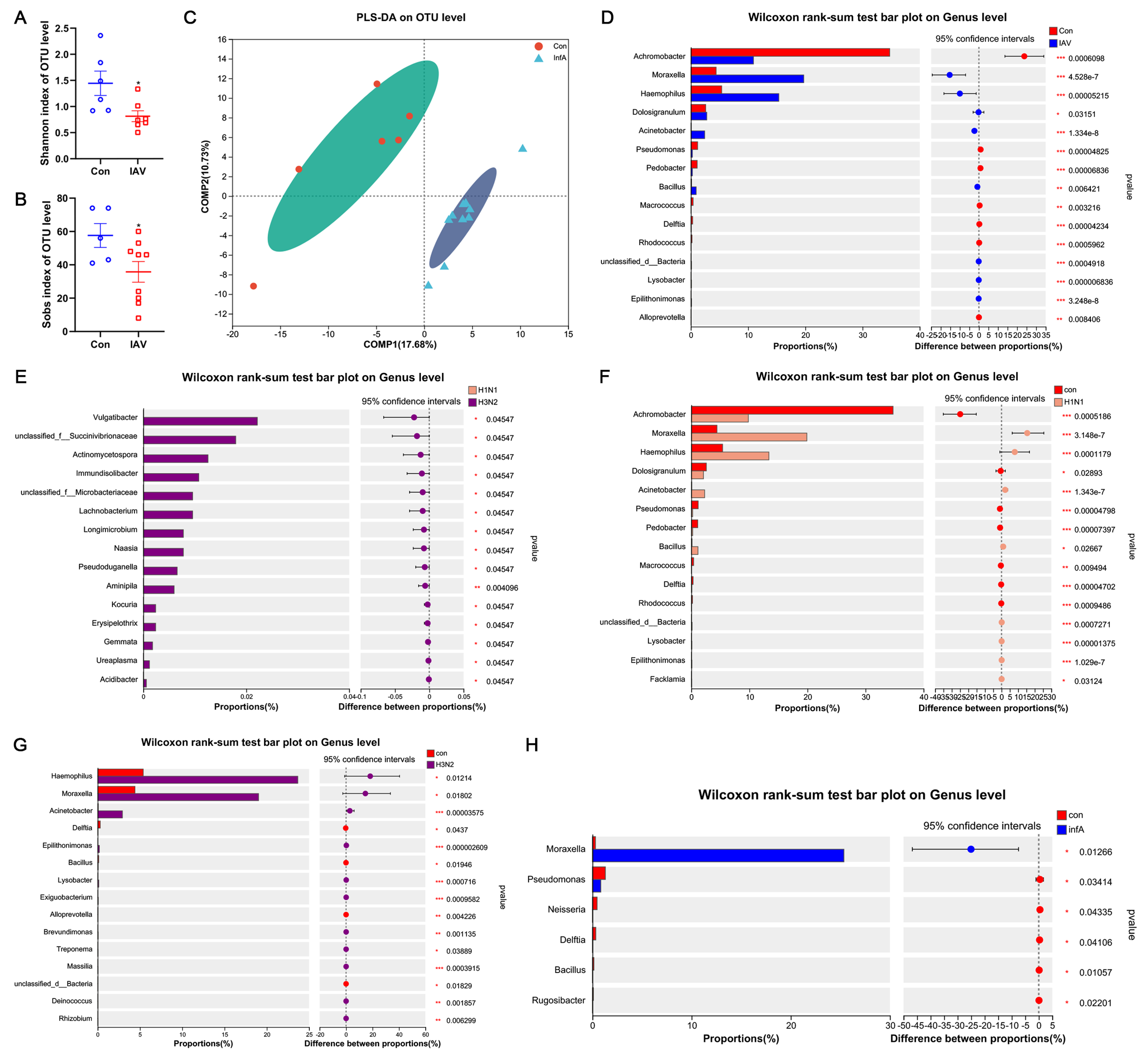

Supplement: Supplementary file 5 [file Image4.jpeg]

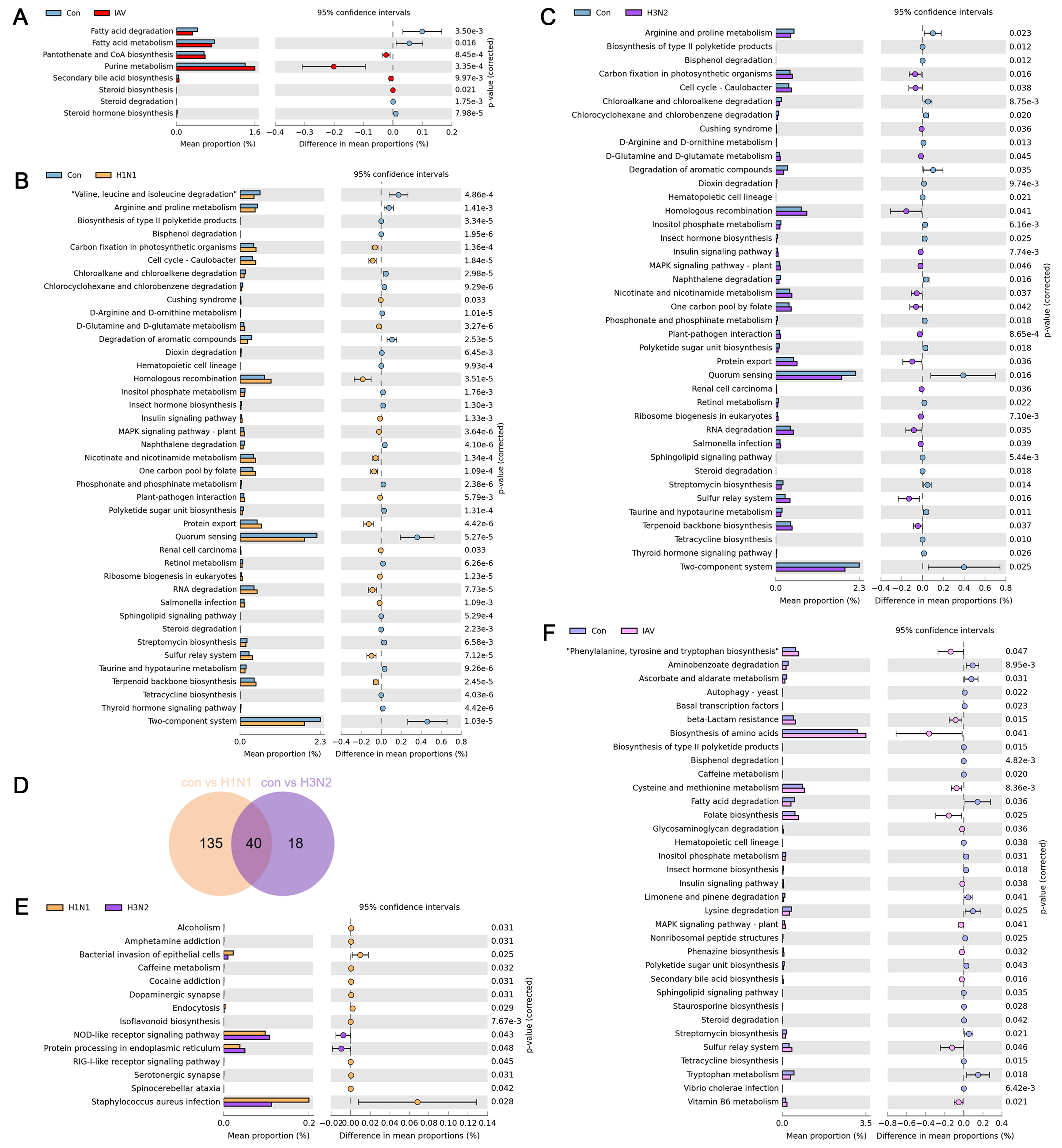

Supplement: Supplementary file 6 [file Image5.jpeg]

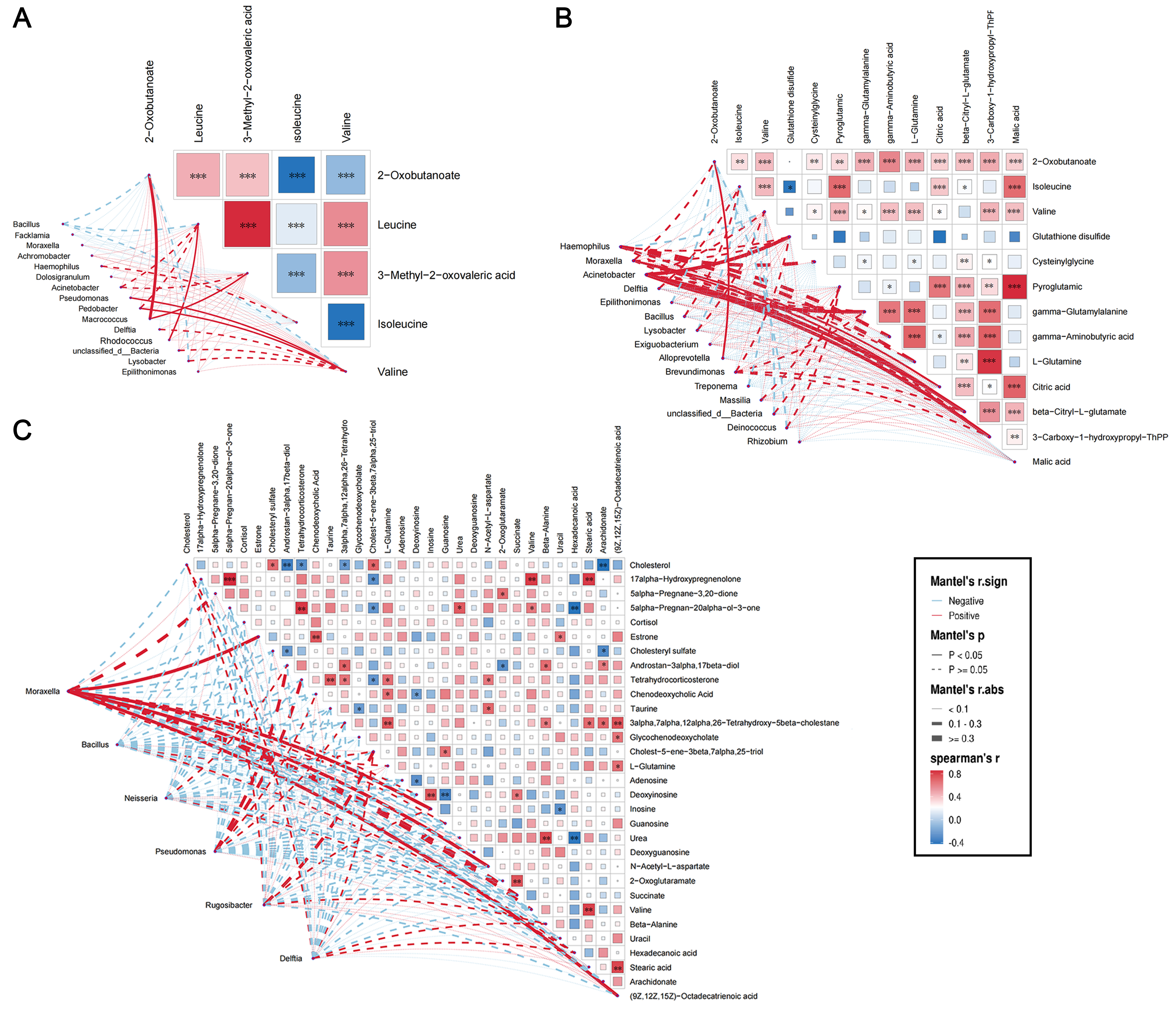

Supplement: Supplementary file 7 [file Image6.jpeg]
